# How Well Do Routine Molecular Diagnostics Detect Rifampin Heteroresistance in Mycobacterium tuberculosis?

**DOI:** 10.1128/JCM.00717-19

**Published:** 2019-10-23

**Authors:** Kamela C. S. Ng, Philip Supply, Frank G. J. Cobelens, Cyril Gaudin, Julian Gonzalez-Martin, Bouke C. de Jong, Leen Rigouts

**Affiliations:** a Mycobacteriology Unit, Department of Biomedical Sciences, Institute of Tropical Medicine, Antwerp, Belgium; b Department of Global Health and Amsterdam Institute for Global Health and Development, Amsterdam UMC, Location Academic Medical Center, Amsterdam, The Netherlands; c Servei de Microbiologia, Centre de Diagnòstic Biomèdic, Hospital Clínic, Institut de Salut Global de Barcelona, Universitat de Barcelona, Barcelona, Spain; d University Lille, CNRS, Inserm, CHU Lille, Institut Pasteur de Lille, U1019, UMR 8204, Center for Infection and Immunity of Lille (CIIL), Lille, France; e Genoscreen, Lille, France; f Department of Biomedical Sciences, University of Antwerp, Antwerp, Belgium; Carter BloodCare and Baylor University Medical Center

**Keywords:** Deeplex-MycTB, GenoscholarNTM+MDRTBII, GenoTypeMTBDR*plus*v2.0, *Mycobacterium tuberculosis*, XpertMTB/RIF, XpertMTB/RIF Ultra, limit of detection, rifampin heteroresistance, rifampin-resistant tuberculosis

## Abstract

Rifampin heteroresistance—where rifampin-resistant and -susceptible tuberculosis (TB) bacilli coexist—may result in failed standard TB treatment and potential spread of rifampin-resistant strains. The detection of rifampin heteroresistance in routine rapid diagnostic tests (RDTs) allows for patients to receive prompt and effective multidrug-resistant-TB treatment and may improve rifampin-resistant TB control. The limit of detection (LOD) of rifampin heteroresistance for phenotypic drug susceptibility testing by the proportion method is 1% and, yet, is insufficiently documented for RDTs. We, therefore, aimed to determine, for the four RDTs (XpertMTB/RIF, XpertMTB/RIF Ultra, GenoTypeMTBDR*plus*v2.0, and GenoscholarNTM+MDRTBII), the LOD per probe and mutation, validated by CFU counting and targeted deep sequencing (Deeplex-MycTB). We selected one rifampin-susceptible and four rifampin-resistant strains, with mutations D435V, H445D, H445Y, and S450L, respectively, mixed them in various proportions in triplicate, tested them with each RDT, and determined the LODs per mutation type. Deeplex-MycTB revealed concordant proportions of the minority resistant variants in the mixtures. The Deeplex-MycTB-validated LODs ranged from 20% to 80% for XpertMTB/RIF, 20% to 70% for Xpert Ultra, 5% to 10% for GenoTypeMTBDRplusv2.0, and 1% to 10% for GenoscholarNTM+MDRTBII for the different mutations. Deeplex-MycTB, GenoTypeMTBDR*plus*v2.0, and GenoscholarNTM+MDRTBII provide explicit information on rifampin heteroresistance for the most frequently detected mutations. Classic Xpert and Ultra report rifampin heteroresistance as rifampin resistance, while Ultra may denote rifampin heteroresistance through “mixed patterns” of wild-type and mutant melt probe, melt peak temperatures. Overall, our findings inform end users that the threshold for reporting resistance in the case of rifampin heteroresistance is the highest for Classic Xpert and Ultra to resolve phenotypic and genotypic discordant rifampin-resistant TB results.

## Abstract (precis)

Rifampin heteroresistance—where rifampin-resistant and -susceptible tuberculosis (TB) bacilli coexist—may result in failed standard TB treatment and potential spread of rifampin-resistant strains. The detection of rifampin heteroresistance in routine rapid diagnostic tests (RDTs) allows for patients to receive prompt and effective multidrug-resistant-TB treatment and may improve rifampin-resistant TB control.

## INTRODUCTION

Resistance to rifampin (RIF)—the most potent core drug in the standard tuberculosis (TB) treatment regimen (1)—is a major barrier to TB control. In 2017, 71% of global RIF-resistant TB (RR-TB) cases were not diagnosed (2). The diagnosis of RR-TB may be complicated by RIF heteroresistance that is observed in patient samples where RR and RIF-susceptible (RS) strains coexist (3, 4), which may be missed and diagnosed as RS because of detection limits. RIF heteroresistance may arise from an existing resistant clonal subpopulation or from a mixed infection of independent strains with RR and RS profiles. Such heteroresistance, also known as “unfixed” resistance, precedes full-blown resistance (“fixed” resistance, 100% RR) as a result of further resistance selection under treatment (4–7). The failure to detect these minority resistant variants can, thus, result in unsuccessful treatment and potential spread of RR-TB strains (3, 4, 8).

The World Health Organization currently endorses the use of rapid diagnostic tests (RDTs) for the timely detection of RR-TB strains, namely, XpertMTB/RIF (classic Xpert), Xpert Ultra (Ultra), and the line probe assays (LPAs) GenoTypeMTBDR*plus*v2.0 (LPA-Hain) and GenoscholarNTM+MDRTBII (LPA-Nipro) (9–11). Among these RDTs, only the LPAs are currently known to explicitly detect RIF heteroresistance in the case of mixtures with mutations covered by the assay, which is exemplified by both wild-type (WT) and mutant bands being present, also known as “mixed patterns” (4, 12).

In this study, we define RIF heteroresistance limit of detection (LOD) as the minimum proportion of mutant bacilli in a total mycobacterial population present in a sample that is needed for RIF resistance to be detected (4). It is known that phenotypic drug susceptibility testing (DST) by the proportion method determines at least 1% resistant subpopulation in clinical samples (4, 13, 14). In the above-mentioned RDTs, however, the RIF heteroresistance LOD in association with the specific *rpoB* mutation is insufficiently documented. In the case of classic Xpert, previous studies report LOD values ranging from 65% to 100% (15, 16); for Ultra, the first validation study conducted by the manufacturer presented LODs only for mutations L430P, H445N (20% to 40%), and S450L (5% to 10%) (17); whereas LODs of GenoscholarNTM+MDRTBII have not been reported yet.

High coverage depths achieved through preselected amplified genes allow targeted deep sequencing to capture and quantify minority resistant variants of Mycobacterium tuberculosis mutants and detect RIF heteroresistance with high sensitivity (18, 19). As an example of such an approach, Deeplex-MycTB (Deeplex; Genoscreen, France) uses ultradeep sequencing of a single, 24-plexed amplicon mix to detect drug resistance-associated mutations in M. tuberculosis complex (MTBC) strains, in addition to mycobacterial species identification and MTBC strain genotyping, with a 24- to 48-h turnaround time, starting from smear-positive clinical samples or primary cultures. Among the 18 main gene targets associated with first- and second-line drug resistance included in Deeplex-MycTB, the *rpoB* gene, associated with RR, is covered by two amplicons, of which one comprises the main mutation hot spot region, also known as the RIF resistance-determining region (RRDR) (20).

Precise documentation of LODs for the most relevant *rpoB* mutations and for the state-of-the-art RDTs is necessary for timely and more accurate identification of RIF heteroresistance and prompt initiation of appropriate treatment. Therefore, we determined the thresholds of classic Xpert, Ultra, LPA-Hain, and LPA-Nipro for detecting RIF heteroresistance linked with RR mutations S450L, D435V, H445D, and H445Y, in relation with the different probes used in each RDT. These four mutations are most frequently detected in association with RR-TB in the global MTBC strain population, according to large-scale studies (10, 21). We used simulated mixtures of selected, cultured RR and RS-TB strains, at ratios initially based on CFU counts after McFarland standardization of the bacillary suspensions. Targeted deep sequencing by Deeplex-MycTB was used as a reference for the quantitative assessment of the RR:RS ratios.

## MATERIALS AND METHODS

### Selection of strains.

We selected one RS and four RR M. tuberculosis strains from the Belgian Coordinated Collection of Microorganisms hosted in the Institute of Tropical Medicine Antwerp (22), on the basis of the presence of mutations confirmed by Sanger sequencing and captured by the mutation probes of LPA-Hain and LPA-Nipro, namely, the Beijing 2.2.1.1 strains TB-TDR-0090 (ITM-041208 with *rpoB* mutation S450L [S531L in Escherichia coli numbering]) and TB-TDR-0100 [ITM-041220, D435V (D516V)] and the LAM 4.3.4.2 strains TB-TDR-0036 [ITM-000930, H445D (H526D)] and TB-TDR-0131 [ITM-041289, H445Y (H526Y)] (23). The RS strain was the Euro-American lineage 4.9 TDR-0140 (ITM-091634, *rpoB* wild type).

### Bacillary suspensions and baseline CFU counting.

We prepared two batches of McFarland standard 1 (24, 25) suspensions for each RR and the RS strain. To check if numbers of bacilli were similar among the cultures after McFarland standardization, we performed CFU counting by spread plating of serial dilutions until 10^−4^ to 10^−6^ (see Fig. S1a in the supplemental material). From each dilution, 100 μl was plated in triplicate onto Dubos agar plates that were sealed with a double layer of parafilm, placed in ziplock bags, and incubated at 37°C for 4 weeks before colony counting. The first batch was used to prepare RR:RS mixtures for testing by classic Xpert, LPA-Hain, and LPA-Nipro, and the second batch was prepared for assessment by Ultra, which was only released after initial testing.

### Simulation of RIF heteroresistance.

RIF heteroresistance was simulated for each mutation type by mixing McFarland standard 1 suspensions of the RS and respective RR strains in triplicate in the proportions (R:S) 0:100, 1:99, 5:95, 10:90, 20:80, 30:70, 40:60, 50:50, 60:40, 70:30, 80:20, and 100:0 (Fig. S1b) and vortexing the mixtures for 20 seconds. Replicate 3 of each RR:RS mixture per batch was tested by targeted deep sequencing (Deeplex-MycTB), the results of which served for cross-validation of variant quantification.

### Subjection of RR:RS mixtures to RDTs.

We subjected mixtures of RR- and RS-TB bacillary suspensions to classic Xpert and Ultra testing (10, 11) and thermolysates to LPA-Hain and LPA-Nipro following the manufacturers’ instructions. We recorded the LODs and corresponding RDT probe reactions per mutation type, in comparison with values cross-validated by CFU counts and variant quantification with Deeplex-MycTB.

The first reading of the LPA strips was done by the person who prepared the mixtures and performed the tests, while the second reading was done by a colleague who was blind to the sample information to ensure an objective reading of raw results. Additionally, for LPA-Nipro, the Genoscholar Reader, a mobile application developed by Nipro (Osaka, Japan), was utilized.

**FIG 1:**
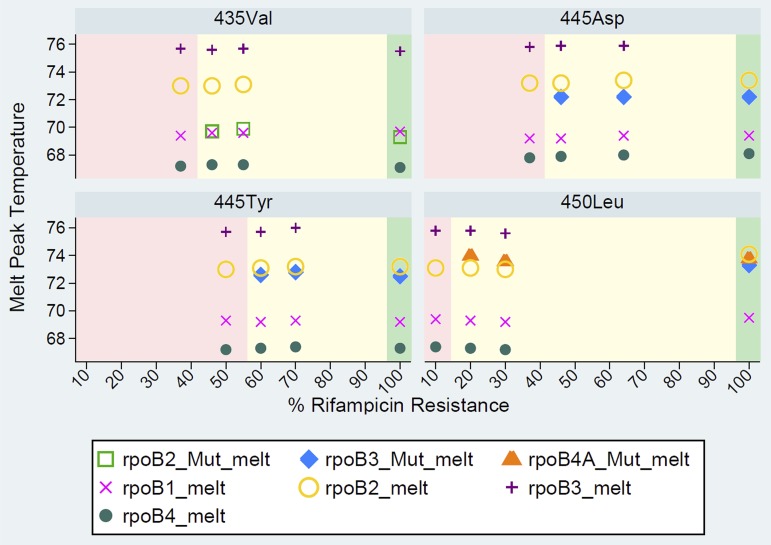
Presence/absence of Xpert Ultra wild-type and mutant melt probes detected for the rifampin-resistant (RR):rifampin-susceptible (RS) mixtures per mutation type at various proportions of the minority resistant variants at the limit of detection (LOD) reported as heteroresistant (shaded in yellow), below LOD reported as RS (shaded in pink), and at full RR reported as resistant (shaded in green).

In their standard reporting, Classic Xpert and Ultra (see Fig. S4a in the supplemental material) report RIF heteroresistance, above or equal its LOD, as RR. Users can indirectly infer RIF heteroresistance from Ultra data, as shown by the simultaneous presence of WT and mutant (Mut) melt peak temperatures at the “melt peaks” tab (Fig. S4b). When generating the results in portable document format, users must tick the melt peaks box to include the melt peak temperatures associated with each WT and Mut melt probe, which may denote RIF heteroresistance in the extended report (Fig. S4b). Full resistance is detected by the presence of both rpoB4A and rpoB3 Mut melt probes for mutation S450L, whereas RIF heteroresistance is detected only by the rpoB4A Mut melt probe in combination with the corresponding WT melt probe (Fig. 1).

### Quantitative assessment of mutant proportions by Deeplex-MycTB.

Per batch, we quantitatively assessed proportions of resistant subpopulations in replicate 3 of the prepared RR:RS mixtures by Deeplex-MycTB. These proportions were determined by calculating the mean percentages of minority resistant variants across all mutation positions borne by each of the RR strains in the *rpoB* gene and other gene targets, depending on the strain genetic background.

Thermolysates of the RR:RS mixtures prepared as previously described (26) were subjected to amplicon deep sequencing, using Deeplex-MycTB kits for the amplification according to the manufacturer’s instructions. Replicates 3 of all first batch mixtures were tested with the classical 18-gene target version, while replicate 3 of the second batch with S450L-WT mixtures was tested with a customized version, including 5 gene targets (*rpoB*, *katG*, *inhA*, *fabG1*, and *gyrA*). Amplicons were purified using Agencourt AMPure XP magnetic beads (Beckman Coulter, USA) and quantified by fluorescence quantification in 96-well plates on a Victor instrument. Paired-end libraries of a 150-bp read length were prepared using the Nextera XT DNA sample preparation kit (Illumina, Inc., San Diego, CA, USA) and sequenced on an Illumina MiniSeq instrument using standard procedures. Variant calling was performed using a dedicated, parameterized web application developed by Genoscreen. The nominal threshold for calling minority resistant variants—indicating heteroresistance for drug resistance-associated mutations—is set at a minimum of 3% of all reads, after filtering and depending on the coverage depths, to minimize false-positive calls due to background technical noise (20, 27, 28). Variants present in lower proportions than 3% in the relevant *rpoB* mutation positions were detected separately from the web application (29), without application of this nominal threshold in the analysis pipeline.

## RESULTS

After McFarland standardization of the different strain cultures, the mean CFU counts for dilutions 10^−4^ to 10^−6^ of the three replicates of both first and second batches of the different RS and RR bacillary suspensions were very similar (see Table S1a and b in the supplemental material). The proportions obtained from the Deeplex-MycTB analysis were overall consistent with the expected mixture ratios and the relative variation seen among CFU counts, as relative deviations from expected values were limited to 0% to 16% (Table 1; see Fig. S2 in the supplemental material).

**TABLE 1:**
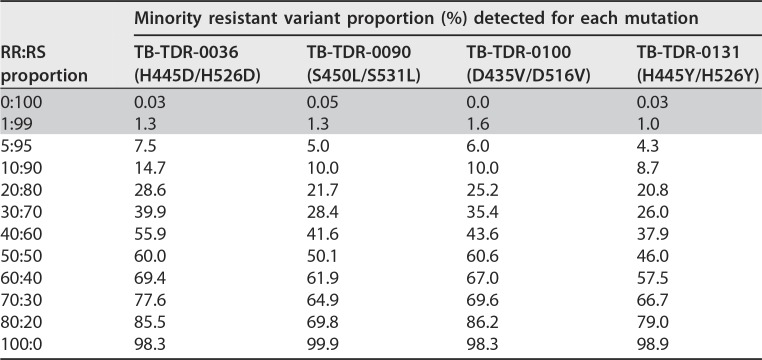
Proportions of minority resistant variants detected among the rifampin-resistant:rifampin-susceptible mixtures by targeted deep sequencing^*a*^

a The values represent the percentage of sequence reads bearing the indicated *rpoB* mutation. Gray boxes represent proportions below the nominal threshold of 3% for calling minority resistant variants used in the Deeplex-MycTB application.

We excluded replicate 3 from the first batch of the S450L-WT preparation due to a substantially high deviation across all mixture ratios, as revealed by Deeplex-MycTB (see Fig. S3 in the supplemental material). This deviation potentially reflects pipetting variation or bacillary clumping despite similar CFU counts of the respective RR and RS strains. Hence, for the S450L-WT mixture, replicate 3 of batch 2, which was initially only tested by Ultra and Deeplex, was also subjected to classic Xpert and the LPAs, given the good correlation between the two batches and to ensure triplicate testing for all mixtures.

In line with these levels of experimental variation, all *rpoB* variants from 5:95 RR:RS mixtures were called by the Deeplex application in proportions ranging from 4.3% (H445Y) to 7.5% (H445D) (Table 1). When the analysis pipeline was used without applying this threshold, expected variants of 1:99 mixtures were also detected in percentages ranging from 1.0% (H445Y) to 1.6% (D435V), which were above background levels of 0.0% to 0.05% detected in the 0:100 mixtures (i.e., WT strain only) on these specific sequence positions. The average minimum coverage depth observed from the Deeplex analyses was 1,595 reads.

Among the available classical RDTs, LPAs had a lower LOD to detect RIF heteroresistance than classic Xpert and Ultra. The proportion of variants required to be detectable through LPA-Hain was 5% for mutation S450L and 5% to 10% for mutations D435V, H445D, and H445Y (see Table S2 in the supplemental material). LPA-Nipro performed similarly, with 1% to 5%, 5%, 5% to 10%, and 10% resistant bacilli detected for mutations S450L, H445D, D435V, and H445Y, respectively (Table S2). LPA reading results were consistent between the two readers. Additionally, the “automated” Genoscholar reader had similar results as the manual reading for mutations H445Y and S450L, whereas it had lower sensitivity for mutations D435V (20% Genoscholar reader versus 5% manual reading) and H445D (10% Genoscholar reader versus 5% manual reading) (see Table S3 in the supplemental material; see Fig. S5a through d in the supplemental material).

In contrast, classic Xpert detected mutation S450L only in mixtures with at least 20% to 40% resistant bacilli, mutation H445D with at least 40% to 60%, and mutations D435V and H445Y with at least 70% to 80% mutant bacilli (Table S2). Likewise, Ultra required a minimum of 20% to 30% resistant bacilli to detect mutation S450L, 40% to 50% for D435V, 40% to 60% for H445D, and 60% to 70% for H445Y (Table S2; Fig. 1).

Notably, in case of S450L at 20% mutant bacilli, only Ultra rpoB4 Mut melt A probe was observed, whereas both rpoB4 Mut melt A and rpoB3 Mut melt probes were present in the case of 100% S450L (Fig. 1). For mutations D435V, H445D, and H445Y, only one Mut melt probe was observed, whether hetero- or fully resistant. The melt peak temperatures did not differ among hetero- or fully resistant populations for all four mutations tested (Fig. 1).

## DISCUSSION

Consistent with previous studies, we found that LPA-Hain detects RIF heteroresistance better than classic Xpert and Xpert Ultra in samples with sufficient M. tuberculosis complex target DNA and subpopulations that carry the most frequently occurring RR-conferring mutations. The consistent CFU counts (Table S1a and b) and next-generation sequencing (NGS) data from targeted deep sequencing (Table 1, Fig. S2) did not suggest suboptimal preparation of the mixtures, supporting the LODs we observed that differed by capturing RDT probe and mutation type. Clearly, Deeplex-MycTB provided useful quantitative information for validating qualitative observations from the RDTs. We also show here that in contrast with classic Xpert, Xpert Ultra may denote RIF heteroresistance through mixed patterns of WT and mutant melt probe melt peak temperatures, which can be leveraged to inform target end users, such as reference laboratory staff and researchers.

The different LODs observed can be linked to the inherent detecting mechanisms of the RDTs. Xpert is an automated cartridge-based assay that uses a heminested real-time PCR assay and molecular beacon technology in which short overlapping probes bind to the RRDR of the WT M. tuberculosis *rpoB* gene (15, 30). The line probe assays, on the other hand, rely on multiplex amplification and reverse hybridization, involving both WT and mutant probes on a membrane strip (31).

The LODs of LPA-Hain were consistent with a previous study that used version 1 of the LPA-Hain kit for mutations H445Y and S450L (4). The initial visual reading of LPA results was consistent with results of the second reading conducted in a blind manner for both LPA-Hain and LPA-Nipro, and Genoscholar Reader for LPA-Nipro, although we observed that the mobile application was less sensitive than the visual reading for mutations D435V and H445D.

Classic Xpert performed relatively poorly in detecting heteroresistant mixtures with mutation S450L, which is globally by far the most prevalent allele in RR-TB (10). Nevertheless, the 20% to 40% LOD we found for classic Xpert was lower (more sensitive) than that of previous studies, which recorded 65% to 90% LOD (15, 16). Ultra performed similarly to classic Xpert in detecting minority resistant variants of mutations S450L and H445D but did relatively better in capturing those of mutations D435V and H445Y (Table 2). The 20% Ultra LOD for S450L was slightly less sensitive than the 5% to 10% LOD reported for the same mutation by Chakravorty and colleagues (17) who tested single mixtures of RR and RS DNA. We observed higher (less sensitive) LODs for the non-S450L mutations, consistent with their findings.

**TABLE 2:** Limits of detection for rifampin heteroresistance among triplicates^*a*^

Strain ID	Rifampin resistance-conferring mutations		Values by RDT quantified by Deeplex							
			LPA-Hain (%LOD)^1^	Deeplex (%QP)^1^	LPA-Nipro (%LOD)^1^	Deeplex (%QP)^1^	Xpert (%LOD)^1^	Deeplex (%QP)^1^	Ultra (%LOD)^2^	Deeplex (%QP)^2^
TB-TDR-0036	H445D^*b*^	H526D^*c*^	5–10	7.5	5	7.5	40–60	55.9	40–60	
TB-TDR-0090	S450L^*b*^	S531L^*c*^	5	5	1–5	5	20–40	21.7	20–30	21.7
TB-TDR-0100	D435V^*b*^	D516V^*c*^	5–10	6	5–10	6	70–80	69.6	40–50	
TB-TDR-0131	H445Y^*b*^	H526Y^*c*^	5–10	4.3	10	8.7	70–80	66.7	60–70	

a With quantification of the respective lowest proportion by targeted deep sequencing (Deeplex-MycTB). ID, identifier; Deeplex-MycTB, targeted deep sequencing; RDT, rapid diagnostic test; LPA-Hain, GenoTypeMTDBR*plus*v2.0; LPA-Nipro, GenoscholarNTM+MTBII; Xpert, classic XpertMTB/RIF; Ultra, Xpert MTB/RIF Ultra; 1, batch 1; 2, batch 2; %LOD, limit of detection (range) among triplicates for the RDTs; %QP, quantified proportion of minority resistant variants determined by Deeplex on replicate 3.

b In M. tuberculosis numbering.

c In E. coli numbering.

Apart from the slight differences in the LODs recorded for classic Xpert and Ultra, we observed that Ultra allows users to infer from raw results on the computer screen, under the melt peaks tab, the phenomenon of heteroresistance (Fig. S4b; Fig. 1), which is not possible from classic Xpert data. RIF heteroresistance may be rapidly detected by Ultra through observed melt peak temperatures of both WT and Mut melt probes akin to mixed patterns of absent WT and developed mutant bands in LPA. Furthermore, in a sample with the S450L mutation, observing only the melt peak temperature of rpoB4 Mut melt A probe (versus rpoB4 Mut melt A and rpoB3 Mut melt probes for 100% RR) and corresponding WT melt probe may also denote RIF heteroresistance (Fig. 1).

This information is deemed useful for conducting research on Ultra data and could be practical in the field if export of the melt peak temperatures through the LIS port becomes feasible in future software updates, together with other raw data (e.g., melt peak temperatures of WT and Mut melt probes associated with RR mutation). Currently, the raw data of Xpert Ultra are only available directly from the module/computer where it was tested and cannot yet be automatically extracted and shared through the .gxx files. Furthermore, local staff are not usually trained to interpret melt peak temperatures, as it entails considerable effort for the National TB Control Programs (NTPs) to train staff in peripheral settings for such an advanced interpretation of raw results. We have formally requested access from the Xpert Ultra manufacturer for the automated extraction of melt peak temperatures at the central level via nonproprietary connectivity platforms.

Automated capture of the melt peak temperatures by connectivity solutions, such as DataToCare (Savics, Belgium), GXAlert (SystemOne, USA), or C360 (Cepheid, USA), will avoid tedious and error prone manual transcription of the values from the computer screen. It may also allow the laboratory staff and in-country expert Xpert Ultra users to interpret the melt peak temperatures and their association with specific RR mutations, with the support of global expert bodies, such as the Foundation for Innovative New Diagnostics and Global Laboratory Initiative, so that TB reference laboratories can advise peripheral laboratories and clinics optimally. With possible integration into e-Health patient charts beyond TB diagnostics, this will allow the transformation of Xpert Ultra data into usable information for the NTPs using a combination of unique patient identification and geographical information (10, 32). This may not only greatly benefit the remote resolution of discordant results to improve patient management but also aid in building more systematic data on the prevalence and impact of heteroresistance on a programmatic level, which is critical for improving interventions for patients with confirmed heteroresistance.

Our study has limitations. We performed LPA testing of thermolysates in order to allow “optimal” reading of results, as indirect LPA testing increases the intensity of the bands. Routine LPA is commonly done directly on clinical specimens, where background hybridization is more common and can be harder to distinguish from heteroresistance, as both phenomena may produce faint(er) bands which are often disregarded. Thus, the sensitivity for heretoresistance detection that we determined for the LPAs likely represents the upper bound of values achievable in clinical practice. The inclusion of a culture step, however, may lose minority subpopulations (33), bias the ratio of mutant and WT populations especially if there is fitness loss, and cause delay in obtaining results. Moreover, the LPAs will not report heteroresistance for mutations not covered by a mutant probe and are not recommended for testing paucibacillary smear-negative samples due to their lower sensitivity of detecting M. tuberculosis than with Xpert and culture (34–36).

We addressed the potential variability resulting from bacillary clumping and pipetting by the inclusion of biological replicates, showing differences in LOD of a maximum of two dilutions among replicates with the same mutation.

In conclusion, we report the distinct abilities of LPA-Hain, LPA-Nipro, classic Xpert, and Ultra to detect minority resistant variants representing the most common RR-conferring mutations against the quantitative results of Deeplex-MycTB. The LPAs have more sensitive LODs than classic Xpert and Ultra in samples with sufficient M. tuberculosis complex target DNA, although they report heteroresistance only for the four most common undisputed mutations—D435V, H445D, H445Y, and S450L. For mutations without a confirmatory mutant band such as L452P, RIF heteroresistance cannot be detected, and with a faint intensity of the WT band (31), it may be difficult to distinguish between WT and heteroresistance. Ultra can detect RR and RIF heteroresistance associated with all *rpoB* mutations within the hot spot, albeit requiring a higher proportion of mutant bacilli than LPA. The LPAs and Deeplex-MycTB provide direct information on the occurrence of RIF heteroresistance, whereas Ultra, after informing that RR was detected, may suggest RIF heteroresistance only through additional examination of WT and Mut melt probes and corresponding melt peak temperatures in the raw data on the computer screen or in the generated extended report (Fig. S4b).

The clinical importance of heteroresistance is likely substantial (3), akin to fixed, i.e., 100%, resistance. The proportion method for phenotypic DST, which has been around for over half a century, by design, tests for ≥1% resistant subpopulations (4, 13, 14), with a strong predictive value for poor treatment outcome, at least for the core drugs, e.g., fluoroquinolones (37) and RIF (4). Moreover, samples with ≥5% minority *gyrA*-resistant variants were found to have the same MIC level as that of samples with 100% fluoroquinolone resistance (37, 38), while for *rpoB*, the mycobacterium growth indicator tube phenotypic DST results were similar for samples with ≥5% minority resistant variants and 100% RIF resistance (4). Taken together, this implicates that in samples with resistant subpopulations of at least 5%, classification of heteroresistance as RR, even when less sensitive than the proportion method, is probably key, notwithstanding the lack of direct evidence on clinical impact. Our findings can, thus, inform and guide TB reference laboratory staff, health care providers, and researchers that the threshold for reporting resistance in the case of RIF heteroresistance is the highest for classic Xpert and Ultra to resolve phenotypic and genotypic discordant RR-TB results. Prospective large-scale clinical studies using NGS approaches (3, 8) are necessary to establish the proportion of mutants that predicts a poor outcome of treatment with the specific drug.

## Supplementary Material

Supplemental file 1
