# Supplementary material for: How Well Do Routine Molecular Diagnostics Detect Rifampin Heteroresistance in Mycobacterium tuberculosis?
Source: J Clin Microbiol. 2019 Oct 23;57(11):e00717-19. doi: 10.1128/JCM.00717-19 (PMC6812990; doi:10.1128/JCM.00717-19)
Supplement: Supplemental file 1 [file JCM.00717-19-s0001.pdf]

Supplemental File

Table S1a. CFU plating results of bacterial suspensions subjected to classic Xpert, LPA-Hain, LPA-Nipro (**batch 1)**

**Strain ID (rpoB)**	**CFU/ml Replicate 1**						**CFU/ml Replicate 2**						**CFU/ml Replicate 3**					
	**10-4**		**10-5**		**10-6**		**10-4**		**10-5**		**10-6**		**10-4**		**10-5**		**10-6**	
	**range**	**ave**	**range**	**ave**	**range**	**ave**	**range**	**ave**	**range**	**ave**	**range**	**ave**	**range**	**ave**	**range**	**ave**	**range**	**ave**
TDR 36 (H445D)	(9,11)	10	(0,2)	1	(0,0)	0	(8,14)	11	(0,3)	2	(0,0)	0	(6,15)	9	(1,2)	1	(0,0)	0
TDR 140 (WT)	(8,11)	10	(0,2)	1	(0,0)	0	(11,13)	12	(1,2)	1	(0,0)	0	(9,13)	11	(0,2)	1	(0,0)	0
TDR 90 (S450L)	(10,13)	12	(1,4)	2	(0,1)	0	(9,16)	13	(0,3)	2	(0,1)	0	(10,14)	12	(0,3)	2	(0,1)	0
TDR 140 (WT)	(9,11)	10	(1,2)	1	(0,0)	0	(10,11)	11	(1,3)	2	(0,0)	0	(10,12)	11	(0,2)	1	(0,0)	0
TDR 100 (D435V)	(9,11)	10	(0,1)	1	(0,0)	0	(9,13)	11	(0,2)	1	(0,0)	0	(9,14)	11	(0,3)	1	(0,1)	0
TDR 140 (WT)	(11,13)	12	(1,3)	2	(1,1)	1	(9,14)	12	(0,2)	1	(0,0)	0	(9,15)	11	(0,3)	1	(0,0)	1
TDR 131 (H445Y)	(8,9)	9	(0,1)	1	(0,0)	0	(9,12)	10	(0,1)	0	(0,0)	0	(8,10)	9	(0,1)	1	(0,1)	0
TDR 140 (WT)	(10,11)	10	(1,2)	2	(0,0)	0	(11,15)	12	(1,3)	2	(0,0)	0	(10,12)	11	(2,2)	2	(0,0)	0

*shaded replicate was tested in Deeplex Myc-TB

Table S1b. CFU plating results of bacterial suspensions subjected to Ultra (**batch 2)**

**Strain ID (rpoB)**	**CFU/ml Replicate 1**						**CFU/ml Replicate 2**						**CFU/ml Replicate 3**					
	**10-4**		**10-5**		**10-6**		**10-4**		**10-5**		**10-6**		**10-4**		**10-5**		**10-6**	
	**range**	**ave**	**range**	**ave**	**range**	**ave**	**range**	**ave**	**range**	**ave**	**range**	**ave**	**range**	**ave**	**range**	**ave**	**range**	**ave**
TDR 36 (H445D)	(10,12)	11	(1,3)	2	(0,0)	0	(9,11)	10	(1,2)	2	(0,0)	0	(10,11)	11	(1,1)	1	(0,0)	0
TDR 140 (WT)	(9,11)	10	(1,2)	1	(0,0)	0	(10,12)	11	(1,2)	1	(0,0)	0	(10,11)	11	(1,2)	1	(0,0)	0
TDR 90 (S450L)	(11,12)	12	(0,3)	2	(0,0)	0	(12,14)	13	(2,3)	2	(0,0)	0	(13,14)	13	(1,3)	2	(0,1)	0
TDR 140 (WT)	(10,12)	11	(1,2)	1	(0,0)	0	(11,13)	12	(1,3)	2	(0,0)	0	(10,12)	11	(1,2)	1	(0,0)	0
TDR 100 (D435V)	(10,11)	11	(2,3)	2	(0,0)	0	(9,12)	11	(1,2)	1	(0,0)	0	(11,13)	12	(1,2)	1	(0,0)	0
TDR 140 (WT)	(10,12)	11	(1,3)	2	(0,0)	0	(10,13)	12	(0,2)	1	(0,0)	0	(9,15)	11	(1,2)	1	(0,0)	0
TDR 131 (H445Y)	(8,10)	9	(1,2)	1	(0,0)	0	(8,11)	10	(1,2)	1	(0,0)	0	(9,10)	10	(1,1)	1	(0,0)	0
TDR 140 (WT)	(9,11)	10	(1,2)	1	(0,0)	0	(10,12)	11	(1,3)	2	(0,0)	0	(10,12)	11	(0,1)	1	(0,0)	0

*shaded MUT-WT mixtures were tested in Deeplex Myc-TB

Table S2. Lowest percentages of mutant bacilli that were identified by the RDTs as rifampicin heteroresistant. The last column shows the quantified mean percentages of the mutants determined by targeted deep sequencing (Deeplex-MycTB) as reference.

Strain ID (*rpoB*)	LPA-Hain				LPA-Nipro				Classic Xpert			Ultra		
	**LOD range among replicates (% mutant bacilli)**	**WT probe covering muted region (+)**	**MUT probe for relevant mutation (+)**	**Deeplex result for replicate 3**	**LOD range among replicates (% mutant bacilli)**	**WT probe covering muted region (+)**	**MUT probe for relevant mutation (+)**	**Deeplex result for replicate 3**	**LOD range among replicates (% mutant bacilli)**	**WT probe covering muted region (+)**	**Deeplex result for replicate 3**	**LOD range among replicates (% mutant bacilli)**	**WT probe covering muted region (+)**	**Deeplex result for replicate 3**
**TDR 36 (H445D)**	5-10	WT7	MUT2B	7.5	5	S4	R4b	7.5	40-60	D (delayed)	55.9	60	rpoB3	69.4
**TDR 90 (S450L)**	5	WT8	MUT3	5	1-5	S5	R5	5	20-40	E (delayed)	21.7	20-30	rpoB4A	21.7
**TDR 100 (D435V)**	5-10	WT3, WT4	MUT1	6	5-10	S2	R2	6	70-80	B (delayed)	69.6	40-50	rpoB2	43.6
**TDR 131 (H445Y)**	5-10	WT7	MUT2A	4.3	10	S4	R4a	8.7	70-80	D (delayed)	66.7	60-70	rpoB3	57.5

Table S3. Comparison of **Genoscholar Reader** and manual reading results on replicate 3 of RR:RS mixtures

	**LOD (minimum % mutant bacilli for rifampicin resistance (RR) detection)**	
**Strain (mutation)**	**Genoscholar Reader**	**Manual reading**
TDR 36 (H445D)	10	5
TDR 90 (S450L)	5	5
TDR 100 (D435V)	20	5
TDR 131 (H445Y)	10	10

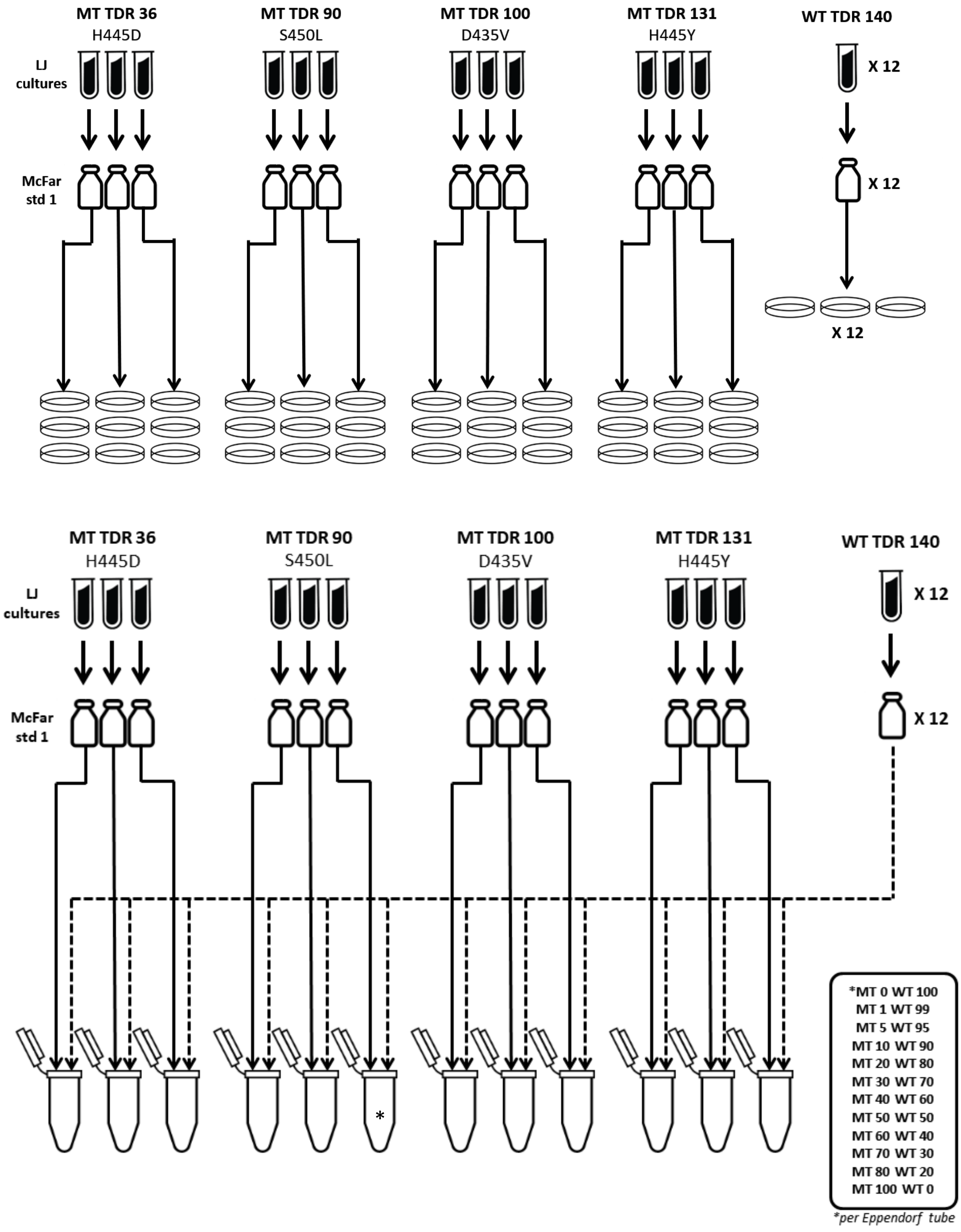

Figure S1 a and b. Experimental design

*tube excluded from the analysis

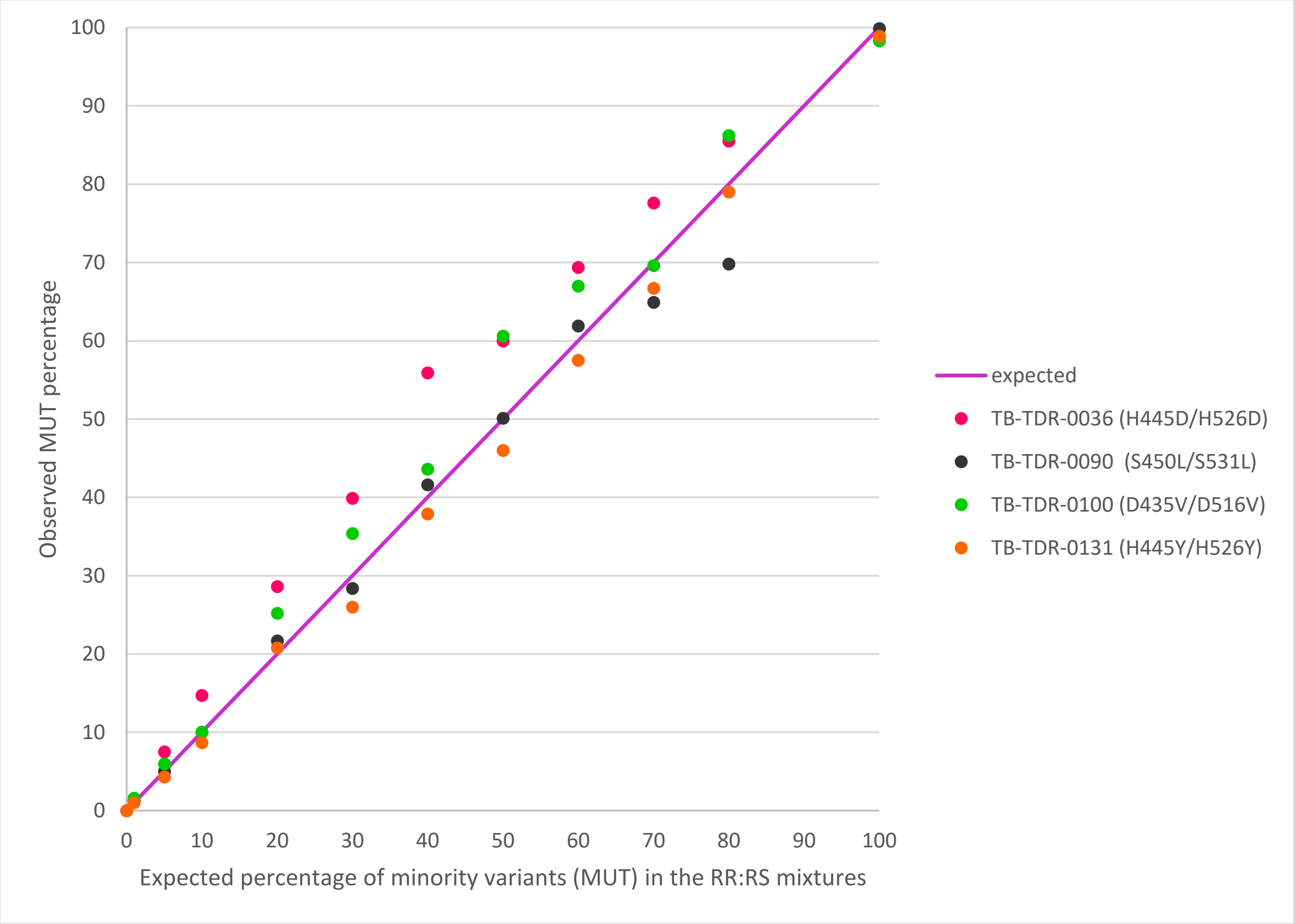

Figure S2. Minority resistant variant proportions detected across all mutation positions by targeted deep sequencing (Deeplex-MycTB) among the rifampicin-resistant:rifampicin-susceptible mixtures. The legend shows the RR-TB strain numbers and the corresponding RR-conferring mutations in *Mycobacterium tuberculosis* / *Escherichia coli* numbering system.

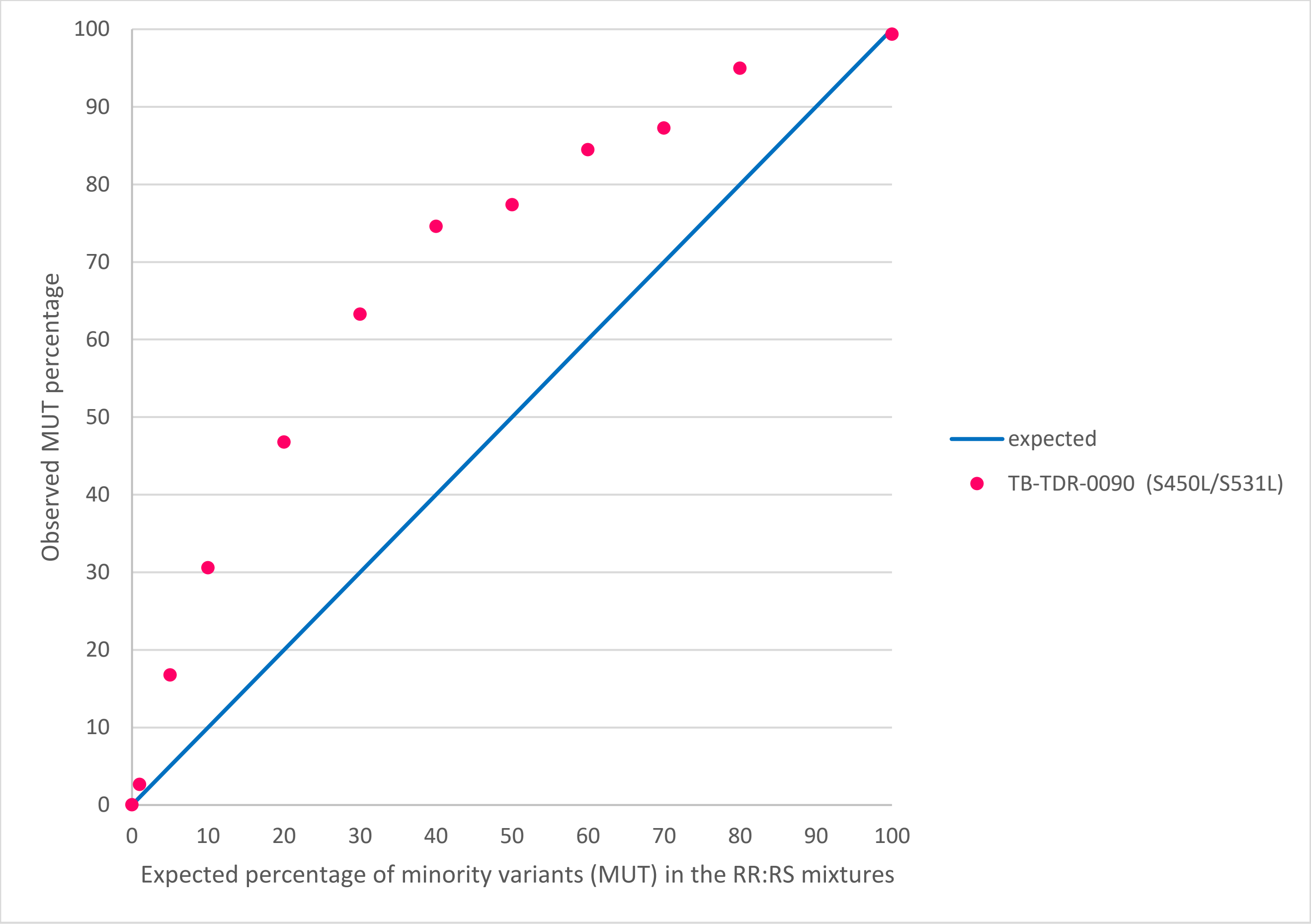

Figure S3. Minority resistant variant proportions detected across all mutation positions by targeted deep sequencing (Deeplex-MycTB) among the rifampicin-resistant:rifampicin-susceptible mixtures of the excluded replicate with mutation Ser450Leu. The legend shows the strain number and RR-conferring mutation in *Mycobacterium tuberculosis* / *Escherichia coli* numbering system.

Assay Information

Assay	Assay Version	Assay Type
Xpert MTB-RIF Ultra	2	In Vitro Diagnostic

Test Result: MTB DETECTED MEDIUM;
RIF Resistance DETECTED

Analyte Result

Analyte Name	Ct	EndPt	Analyte Result	Probe Check Result
SPC	38.9	35	NA	PASS
IS1081-IS6110	16.4	409	NA	PASS
rpoB1	20.6	334	POS	PASS
rpoB2	20.2	219	POS	PASS
rpoB3	24.4	99	POS	PASS
rpoB4	25.0	99	POS	PASS

Figure S4 a. Screenshot of a standard report generated by the Xpert Ultra software

Melt Peaks

Analyte Name	Melt Peak Temperature	Melt Peak Height
rpoB1 melt	69.3	80.4
rpoB2 melt	73.2	120.5
rpoB3 melt	75.8	38.0
rpoB4 melt	67.8	76.1
rpoB1 Mut melt		
rpoB2 Mut melt		
rpoB3 Mut melt	72.2	12.9
rpoB4 Mut melt A		
rpoB4 Mut melt B		

b. Screenshot of an extended report, provided that the ‘Melt Peaks’ box was ticked during generation of results as portable document format

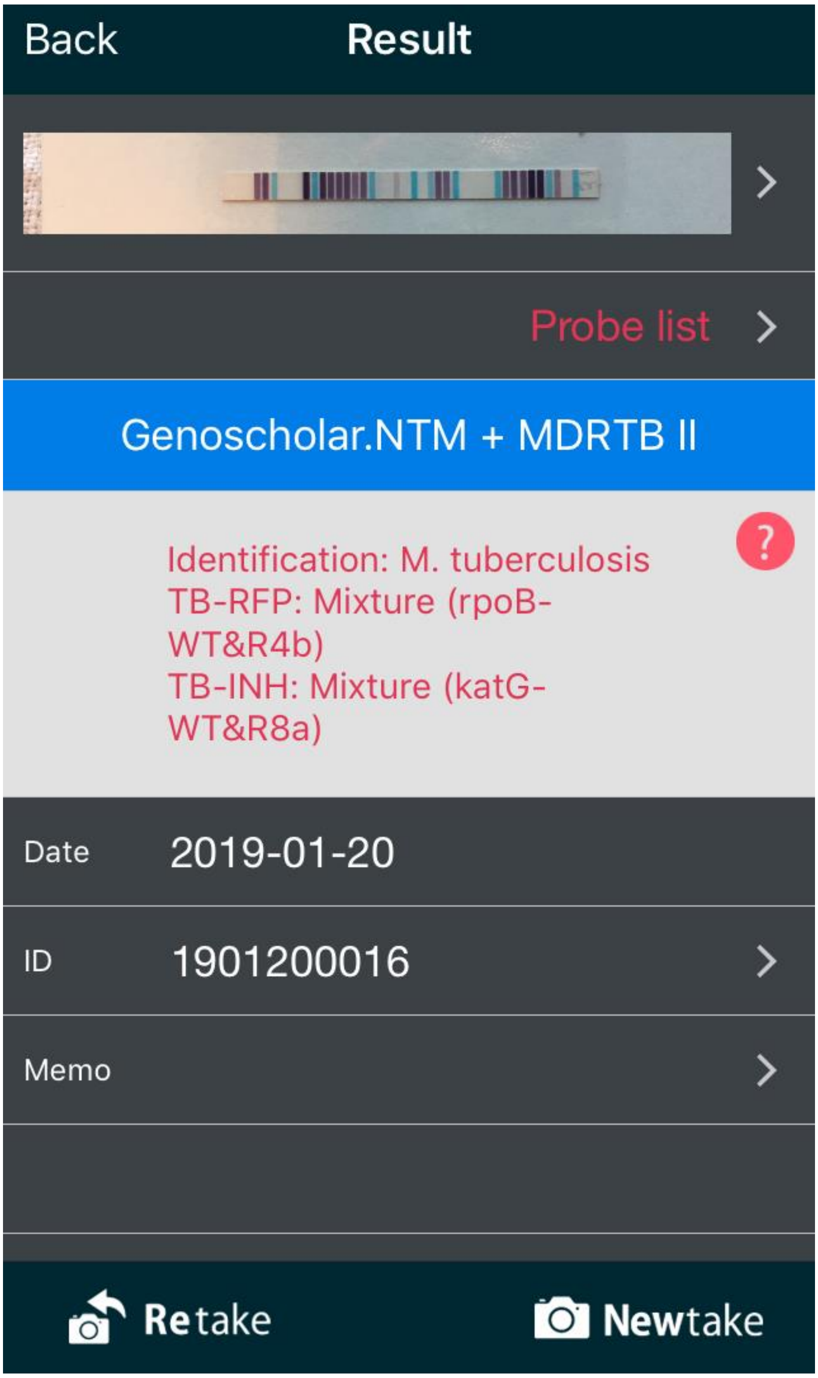

Figure S5 **a.** Screenshot from the Genoscholar Reader application tested via iPhone, at the limit of detection (LOD: 5% minority resistant variants) of GenoscholarNTM·MTBDR II (LPA-Nipro) for RR-TB strain TDR 36 with mutation H445D

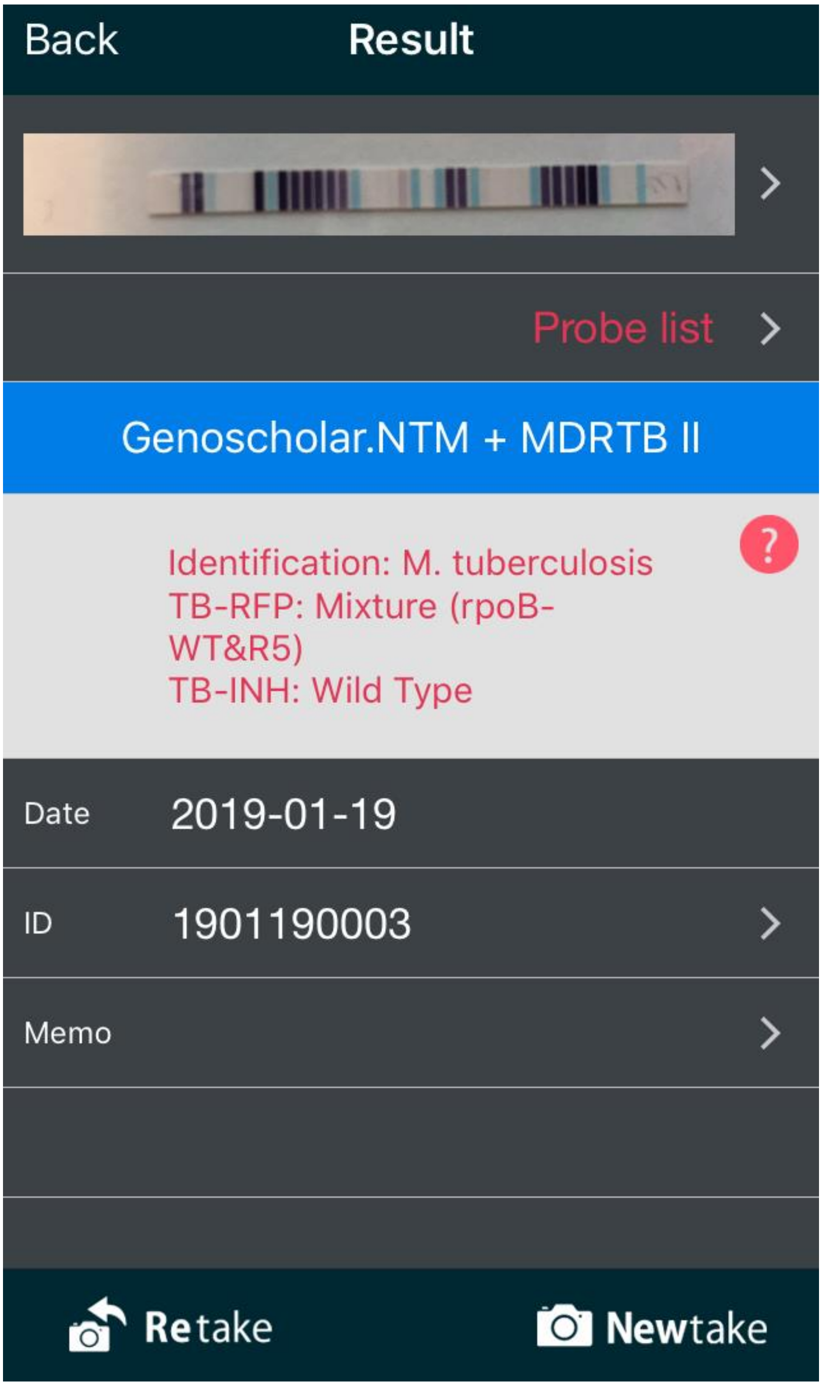

b. Screenshot from the Genoscholar Reader application tested via iPhone, at the limit of detection (LOD: 5% minority resistant variants) of GenoscholarNTM·MTBDR II (LPA-Nipro) for RR-TB strain TDR 90 with mutation S450L

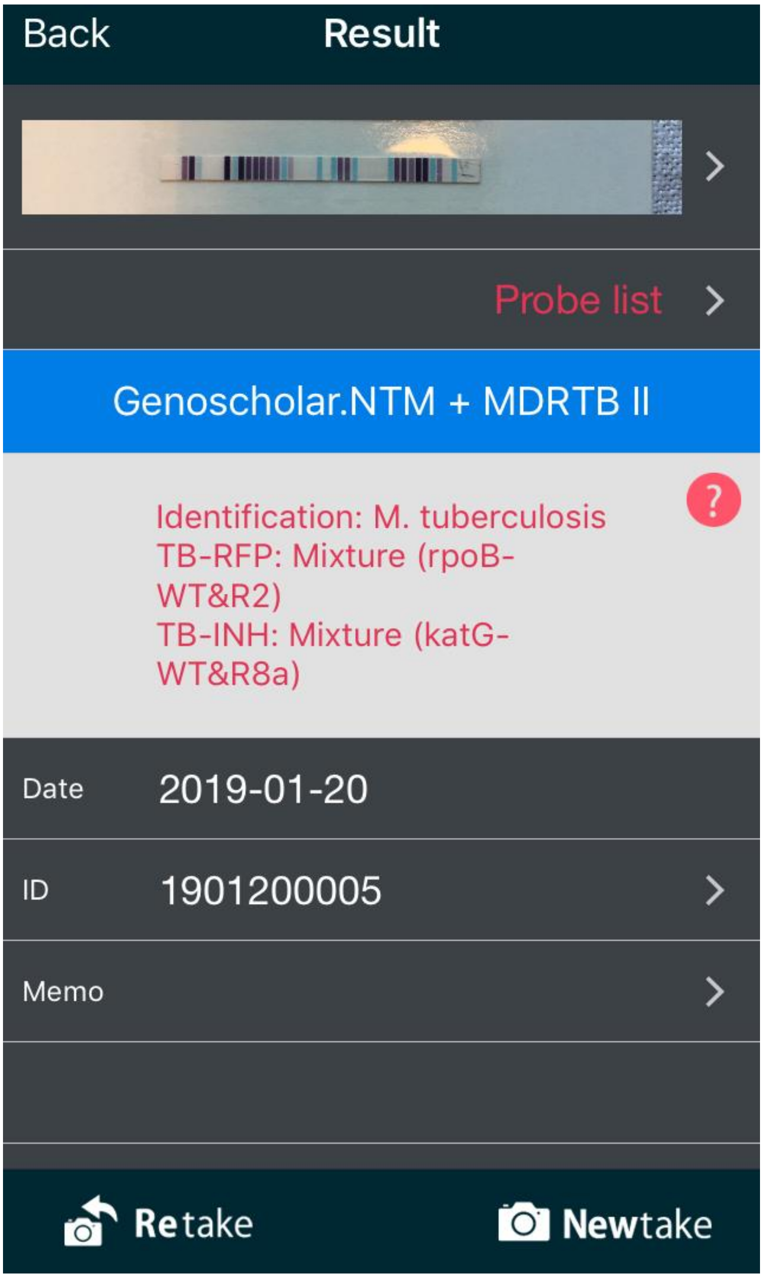

c. Screenshot from the Genoscholar Reader application tested via iPhone, at the limit of detection (LOD: 5% minority resistant variants) of GenoscholarNTM·MTBDR II (LPA-Nipro) for RR-TB strain TDR 100 with mutation D435V

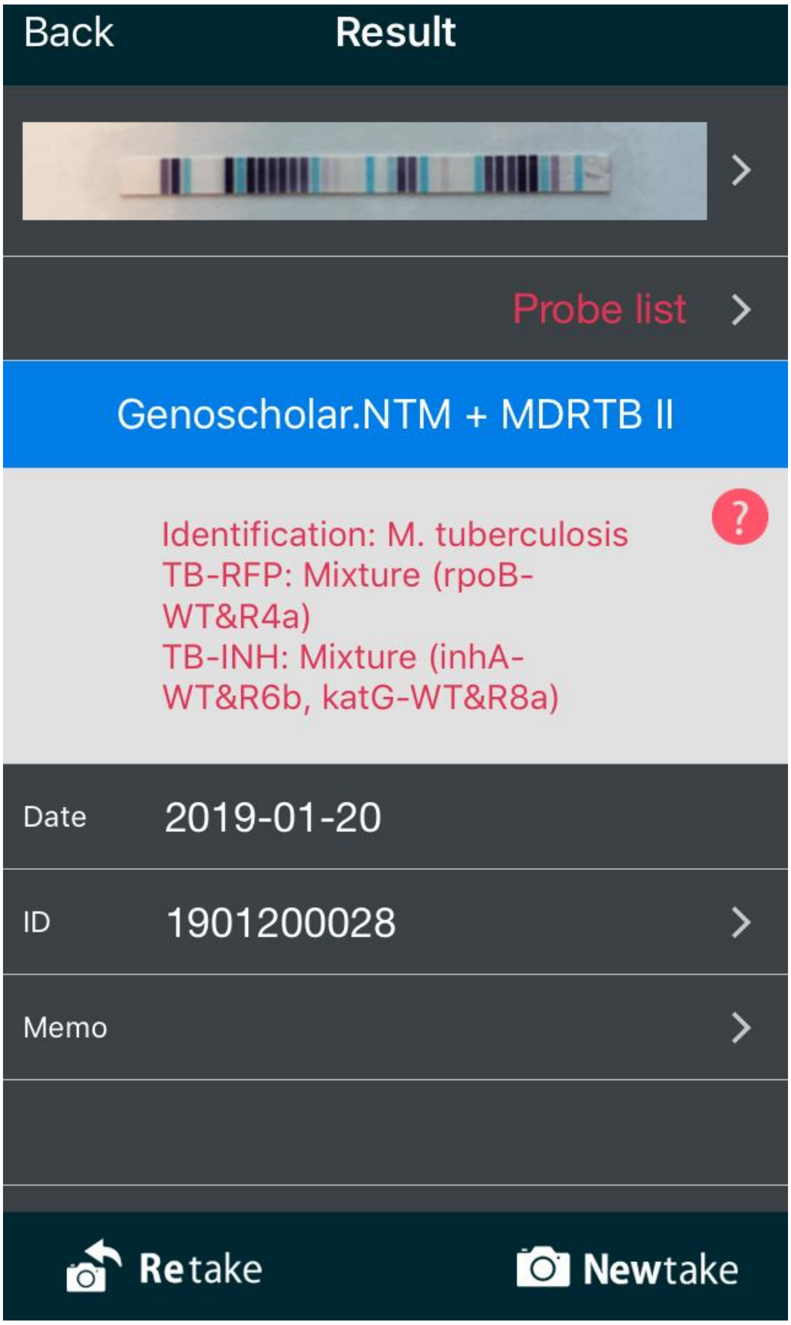

d. Screenshot from the Genoscholar Reader application tested via iPhone, at the limit of detection (LOD: 10% minority resistant variants) of GenoscholarNTM·MTBDR II (LPA-Nipro) for RR-TB strain TDR 131 with mutation H445Y
